# Angiopoietin 2 signaling plays a critical role in neural crest cell migration

**DOI:** 10.1186/s12915-016-0323-9

**Published:** 2016-12-15

**Authors:** Mary Cathleen McKinney, Rebecca McLennan, Paul M. Kulesa

**Affiliations:** 1 Stowers Institute for Medical Research, 1000 E. 50th St., Kansas City, MO 64110 USA; 2 Department of Anatomy and Cell Biology, University of Kansas School of Medicine, Kansas City, KS 64157 USA

**Keywords:** Chick, Cranial, Neural crest, Migration, Angiopoietin 2, Endothelial cells, Transgenic quail

## Abstract

**Background:**

Collective neural crest cell migration is critical to the form and function of the vertebrate face and neck, distributing bone, cartilage, and nerve cells into peripheral targets that are intimately linked with head vasculature. The vasculature and neural crest structures are ultimately linked, but when and how these patterns develop in the early embryo are not well understood.

**Results:**

Using in vivo imaging and sophisticated cell behavior analyses, we show that quail cranial neural crest and endothelial cells share common migratory paths, sort out in a dynamic multistep process, and display multiple types of motion. To better understand the underlying molecular signals, we examined the role of angiopoietin 2 (Ang2), which we found expressed in migrating cranial neural crest cells. Overexpression of Ang2 causes neural crest cells to be more exploratory as displayed by invasion of off-target locations, the widening of migratory streams into prohibitive zones, and differences in cell motility type. The enhanced exploratory phenotype correlates with increased phosphorylated focal adhesion kinase activity in migrating neural crest cells. In contrast, loss of Ang2 function reduces neural crest cell exploration. In both gain and loss of function of Ang2, we found disruptions to the timing and interplay between cranial neural crest and endothelial cells.

**Conclusions:**

Together, these data demonstrate a role for Ang2 in maintaining collective cranial neural crest cell migration and suggest interdependence with endothelial cell migration during vertebrate head patterning.

**Electronic supplementary material:**

The online version of this article (doi:10.1186/s12915-016-0323-9) contains supplementary material, which is available to authorized users.

## Background

Morphogenesis of the vertebrate head involves the close coordination of multiple cell populations that build tissue structures of the face and neck. One example of this is the intimate patterning of neural crest-derived tissues with vasculature. Neural crest cells are highly migratory, emigrate from the dorsal neural tube to peripheral locations along stereotypical migratory pathways, and contribute to multiple tissues. The neural crest cells contribute to the aortic outflow tract and arterial walls of the large blood vessels in the cardiovascular system [1–4]. Angioblasts and endothelial cells are also highly migratory and undergo angiogenesis and vasculogenesis near the ventrally located dorsal aorta and within the mesoderm through which neural crest cells migrate. Major vascular structures in the vicinity of cranial neural crest cell migration are the perineural vascular plexus around the neural tube and branchial arch arteries. Several birth defects affect the form and function of both neural crest-derived craniofacial tissues and head vasculature [2, 5], yet very little is known about the cellular and molecular mechanisms that underlie the complex patterning processes. Thus, studies that examine neural crest and endothelial cell dynamics and underlying molecular choreography would help us better understand vertebrate head patterning.

Details of the spatio-temporal pattern of cranial neural crest and endothelial cell migration remain largely undetermined due to static end-point analyses and lack of an in vivo model system to simultaneously observe the dynamics of both cell populations. Recently, observations in trunk neural crest and endothelial cell dynamics have begun to illustrate the course of events in the trunk, but cranial neural crest cells have yet to be examined [6]. Cranial neural crest cells exit the dorsal neural tube in a rostral-to-caudal manner and migrate along dorsolateral pathways to populate the face and branchial arches [7]. Endothelial cells undergo angiogenesis and vasculogenesis to create the dorsal aorta, perineural vascular plexus, and branchial arch arteries [8–11]. Whether neural crest and endothelial cells share common migratory pathways in space and time and physically interact is unclear. Thus, there is a significant need for dynamic in vivo data that include the simultaneous observation of cranial neural crest and endothelial cell movements in the same embryo during head patterning.

In the absence of dynamic cell behavioral data, it is not surprising that it is largely unknown whether there are distinct molecular signals in the embryonic microenvironment that direct cranial neural crest and endothelial cell movements. We have previously shown that vascular endothelial growth factor (VEGF) is expressed in the chick head ectoderm, directly overlying neural crest cell migratory pathways and in the second branchial arch (BA2) tissue [12]. VEGF is a cranial neural crest cell chemoattractant, and loss of either neuropilin 1 (Nrp1) receptor function on neural crest cells by Nrp1-siRNA or neuropilin 1 signaling by injection of Nrp1-Fc leads to a failure of neural crest cells to invade BA2 [12–14]. VEGF is also a critical regulator of both vasculogenesis and angiogenesis, stimulating endothelial cell proliferation and migration [15, 16]. Thus, the presence of VEGF within the neural crest microenvironment represents an important molecular link between neural crest and endothelial cell dynamics.

We discovered in a previous molecular analysis that migrating chick cranial neural crest cells express high levels of the anti-angiogenic factor angiopoietin 2 (Ang2). Ang2 is a secreted ligand that has been shown in vitro to enhance cell motility in several non-endothelial cell types [17–21]; however, its role in neural crest cell migration has not been studied. In endothelial cells, Ang2 binds to either the Tie1 or Tie2 receptors and is known as a vessel destabilizer that will induce endothelial cell sprouting (in the presence of VEGF) or vessel regression (in the absence of VEGF) [22–26]. Thus, given the presence of VEGF in the cranial neural crest microenvironment, the discovery of Ang2 expression in migrating neural crest cells represents an exciting opportunity to explore its role in neural crest migration interactions during vertebrate head patterning.

In this study, we examined the role of Ang2 on cranial neural crest cell migration using our previously developed in vivo imaging platform [27]. We confirmed Ang2 expression in the chick using multiplexed fluorescence in situ hybridization to simultaneously observe Ang2 mRNA within migrating forkhead box D3 (FoxD3) labeled neural crest cells. We characterized neural crest and endothelial cell dynamics in the same embryo by combining static three-dimensional (3D) and confocal time-lapse imaging of fluorescently marked neural crest cells in Tg(Tie1:H2B-EYFP) quail embryos [28]. By observing and quantifying single cell trajectories, we find that neural crest cell behaviors are dramatically altered in both Ang2 gain- and loss-of-function embryos. We also visualized significant changes in endothelial cell behaviors in Ang2 gain- and loss-of-function embryos. Together, our results offer a unique perspective of neural crest and endothelial cell dynamics in the same embryo and suggest a role for an anti﻿-angiogenic inhibitor to regulate collective neural crest cell migration during vertebrate head patterning.

## Methods

### Embryos and cell labeling

Fertilized leghorn chicken (Centurian Poultry, Lexington, GA, USA) or quail eggs (Ozark Egg Company, Stover, MO, USA) were incubated at 38 °C in a humidified incubator until the desired stages of development [29]. Quail were either wild type or Tg(Tie1:H2B-EYFP) as indicated [28]. Premigratory neural crest cells were labeled by microinjection and electroporation of a DNA plasmid containing a specified fluorescent protein or injection of CellTracker CM-DiI (C-7001, Thermo Fisher) or DiO (V22889, Thermo Fisher) [13] between the 6- and 8-somite stages. For Ang2 gain of function experiments, a vector was created by using a chicken beta-actin promoter driving the cloned Ang2 gene [30] (GenBank NM_204816.1) upstream of an internal ribosome entry site (IRES) sequence and followed by either the mKate2 (Evrogen) or green fluorescent protein (GFP). The GFP control vector is the same but without the Ang2 gene. For Ang2 loss-of-function experiments, potential target sequences to chick Ang2 gene were found using Invitrogen’s target finder that met the criteria outlined in [31] and cloned into the pRFP-RNAiC vector [32]. The empty pRFP-RNAiC vector was used as a control. The efficacy of the short hairpin RNA (shRNA) vectors was chosen based on qPCR analysis of Ang2 on double transfection of chick hepatoma (LMH) cells with Ang2-FL and shRNA. The optimal sequence chosen for Ang2 RNA interference (RNAi) is CTGAGCAGACCCGCAAATTAACAG. For qPCR analysis, approximately 5e5 LMH cells were seeded in wells of a 6-well plate, and single wells were subsequently transfected with either a GFP control, Ang2 full length, or Ang2 full length and Ang2 shRNA. RNA was harvested from each well at 28 h post-transfection and quantified on a NanoDrop 2000 spectrophotometer (Thermo Fisher). From each sample 2 μg of RNA was used to synthesize complementary DNA (cDNA) according to the Applied Biosystems Inc. (ABI) High Capacity cDNA Reverse Transcription kit (4368814, Thermo Fisher). Gene expression analysis was performed on the resulting cDNA by RT-qPCR in technical quadruplicate using TaqMan Gene Expression assays (ANG2: Gg03347361_m1, GAPDH: Gg03346982_m1, and RHOA: Gg03338538_m1) on an ABI7900HT system. No templated negative control reactions were either undetected or produced Ct values more than 10 cycles above any experimental sample. Two reference genes, which were previously shown to have stable expression in LMH cells, were employed for normalization. The data were analyzed with Biogazelle’s qbase software. Expression of Ang2 in each sample is plotted relative to GFP control. Error bars represent the standard error of the mean (SEM). Morpholinos (Gene Tools, Philomath, OR, USA) were suspended in water to 1 mM stock solution and then injected and electroporated into the premigratory neural crest similarly to DNA. Control morpholino sequence CCTCTTACCTCAGTTACAATTTATA and Ang2 sequence CAAGCTGAATCATCAGTGATGCCAT were created with a fluorescein tag for imaging.

Electroporated embryos were incubated 24 h to HH St15 then fixed in 4% paraformaldehyde at 4 °C overnight. Embryos were imaged whole or cut in 100-μm transverse vibratome sections (VT1000S microtome, Leica Biosystems, Buffalo Grove, IL, USA). Cells found inside or outside the majority neural crest pathway (Fig. 5 a–c) were manually counted for comparison. Width measurements were made on 2D projections of whole head images at the top of the otic vesicle (OV) and at the widest part of the stream parallel to the A-P axis of the embryo. A Student’s *t* test was used to compare samples.

#### Original data availability

The datasets generated and/or analyzed during the current study are available as unprocessed raw data in the Stowers Institute for Medical Research original data repository.

### Immunofluorescent labeling

Quail embryos were fixed in 4% paraformaldehyde, embedded in 7% agarose, and vibratome sectioned at 100-μm thickness. The quail monoclonal endothelial cell surface antibody (1:50, QH1, Developmental Studies Hybridoma Bank, RRID:AB_531829, see [33]) and chick neural crest membrane marker HNK1 (1:500, TIB-200 hybridoma cell line, ATCC Cell Lines, RRID: AB_10013722, see [34]) were used to stain the tissue overnight at 4 °C. Secondary antibodies, goat anti-mouse, either Alexa Fluor 546 or 488 for QH1 and HNK1, respectively (1:500, A-21045 RRID: AB_10013722, and A-11030 RRID: AB_2534089, Thermo Fisher), were incubated for 2 h at ambient temperature. Stained sections were imaged by confocal microscopy (Zeiss, LSM 710).

### Fluorescent multiplex in situ hybridization chain reaction (HCR)

Transcripts for Ang2 and FoxD3 were visualized in whole chick embryos by HCR. Embryos were fixed at HH St15 in 4% paraformaldehyde in 0.1% diethylpyrocarbonate (DEPC)-treated phosphate-buffered saline (PBS) at ambient temperature for 2 h, then washed three times in DEPC PBS. The embryos were serially dehydrated in (25%, 50%, 75%, and 100%) methanol and frozen at –20 °C until use (maximum three days). HCR was performed according to the manufacturer’s instructions (Molecular Instruments, California Institute of Technology, Pasadena, CA, USA). After labeling with Alexa Fluor 546 (FoxD3) and Alexa Fluor 647 (Ang2), embryos were cleared in ScaleU2 buffer [35] for at least two days. 3D image sets were collected by confocal microscopy (Zeiss LSM 780), and post-processing was completed in ImageJ. An intensity analysis of the fluorescent signals was performed using an ImageJ plugin (polyline kymograph, Jay Unruh, available at http://research.stowers.org/imagejplugins). A hand-drawn polyline was used to calculate the fluorescence intensity in a 30-pixel-wide area in each channel.

### Embryo time-lapse imaging and analysis

Embryos were fluorescently labeled as described above and allowed to re-incubate to HH St10. Healthy and well-labeled embryos were mounted on Early Chick (EC) culture [27, 36] and placed in a heated, humidified microscope chamber for approximately 30 min to equilibrate. Z-stack confocal images of developing embryos were acquired every 8 min for 8–16 h to be included in our analysis (LSM 710 or 780) using 10–20% 488 nm and 1–4% 561 nm lasers. Post-processing including ImageJ and AutoAligner (Bitplane), and semi-automated cell tracking was completed in Imaris. Mean square displacement (MSD) analysis of cell trajectories was calculated using MATLAB (MathWorks Inc.) and included the msdanalyzer package [37] and MSD Bayes package [38].

### In vitro neural crest cell imaging and analysis

In vitro cultures were prepared similarly to the procedure of [12]. Briefly, neural tubes of stage 9 chick embryos were excised and digested with dispase before plating five half-neural tubes on glass-bottomed MatTek dishes (P35GC-1.5-14-C) coated with 1 mg/mL poly-l-lysine (P7886, Sigma) and 1 mg/mL fibronectin (F1141, Sigma). At 24 h after plating, cells were fixed in 4% paraformaldehyde, and then immunohistochemistry was performed using Phospho-FAK pTyr861 (1:200, 44-626G, Thermo Fisher, RRID:AB_2533703, Lot QJ221024) and HNK1(1:500) primary antibodies with secondary goat anti-rabbit Alexa Fluor 546 (A-11035 RRID: AB_2534093, Thermo Fisher) or goat anti-mouse Alexa Fluor 647 (A-21238 RRID: AB_2535807, Thermo Fisher). We imaged two to four fields of view of four plated neural tubes for each control, Ang2-FL, or Ang2-shRNA condition. The sizes of the phospho-FAK clusters were determined by masking the image with the HNK1 channel and variable size spot detection (Imaris, Bitplane). Spot detection was done with 0.9-μm seed points, and the local region threshold was adjusted so that larger areas were just covered.

### Reverse transcription (RT)-PCR for Ang2

Chick embryos were labeled as described with control GFP expression vector at HH St 6 to 8 somites, then allowed to develop until HH St13. The r4 stream of the neural crest was isolated manually from 12 embryos and dissociated to a single cell suspension. The cell suspension underwent fluorescence-activated cell sorting (FACS) on GFP and Sytox Red (Thermo Fisher) channels and returned 497 cells which were immediately lysed. cDNA was created with the High Capacity cDNA kit (Applied Biosystems). PCR reactions for both beta-actin and Ang2 genes were performed using Ang2 primers GATTTCTGTTCAGACCAACGAG, CAGGATTATCTTAAGAACGTAGC for a 584-bp fragment and beta-actin primers CGGTTTCGCCGGGGACGATG, CGTCAGGTCACGGCCAGCCAGA for a 500-bp fragment.

## Results

To characterize the spatio-temporal aspects of neural crest and endothelial cells during head development, we analyzed HNK1 (neural crest) and QH1 (endothelial cells) antibody-labeled cell positions in fixed quail tissue sections taken from HH St10–15 (HH; Hamburger and Hamilton [29]) embryos. Furthermore, we tracked and quantified individual fluorescently labeled cell behaviors and neural crest-endothelial cell interactions in 3D confocal time-lapse imaging sessions of Tie1:H2B-EYFP transgenic quail whole embryos in explanted cultures as well as neural crest in vitro neural tube cultures. Below, we outline the chronology of the cell positions and cell dynamics data using typical sections from specific developmental stages and individual frames from selected time-lapse imaging sessions.

### Cranial neural crest and endothelial cells arise in distinct locations but move into common spaces

**Fig. 1:**
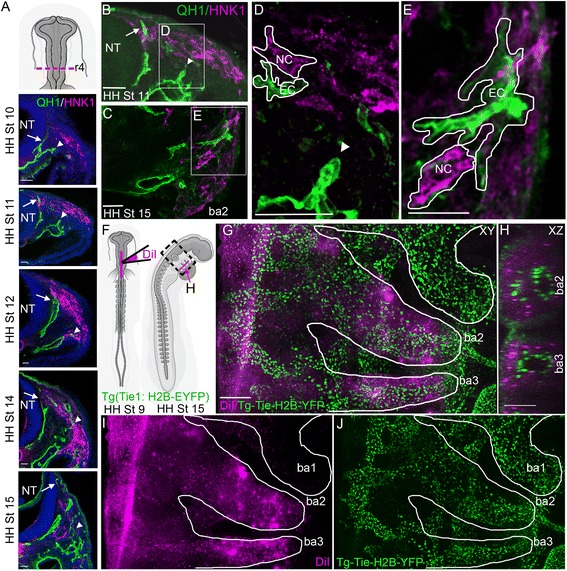
Neural crest and endothelial cells arise in distinct locations but share migratory pathways. **a** *Top*: schematic of head of St10 embryo indicating r4 axial level at which images below were acquired. *Below*: time course of transverse sections of quail at the indicated stages. Endothelial cells, QH1, *green* and neural crest, HNK1, *purple*. Scale bars 40 μm. *Arrows* point to the front of endothelial cells migrating along neural tube, and *triangles* point to endothelial cells branching off dorsal aorta towards neural crest stream. Transverse section of St11 (**b**) and HH St15 (**c**) QH1 and HNK1 stained quail highlighting proximity of neural crest and endothelial cells. *Arrow* highlighting one particularly close sprout to the neural crest stream. Scale bar 50 μm. **d** and **e** zoom in on **b** and **c**, respectively, with pair of close cells *outlined. Arrow head* pointing to endothelial cell sprout moving towards neural crest stream. Scale bar 50 μm. **f** Schematic of neural tube injection of DiI at St10, *left* and imaging at St15, *right. Boxed region* indicates area imaged in **g**–**j**. *Red line* indicates area viewed in **h**. **g** HH St15 stage transgenic quail embryo with DiI marking neural crest in *magenta* and the nuclei of endothelial cells marked in *green*. Scale bar 200 μm. **h** 90^o^ rotated view of BA2 and BA3 (*red line* in **f**) showing the central BA arteries and surrounding neural crest and endothelial cells. Scale bar 100 μm. **i** and **j** Same image as **c** with DiI and YFP channels separated

Pre-otic neural crest cells from the axial level of mid-rhombomere 3 (r3) to mid-r5 exit the dorsal neural tube at HH St10 (Fig. 1a) and follow a dorsolateral pathway just underneath the surface ectoderm. At HH St10, individual endothelial cells appeared scattered throughout the mesoderm, where some vasculogenesis has begun to create rudimentary vessels (Fig. 1a). These vessels stretch around the sides of the neural tube in a ventral-to-dorsal direction in narrow one- to two-cell-wide strands (Fig. 1a, arrows). Endothelial cells that sprouted from the dorsal aorta (DA) formed into multicellular streams that stretched into the branchial arches at HH St11 (Fig. 1a, triangles). An individual stream bifurcated into two branches to position some cells within the neural crest migratory stream and other cells into the branchial arches.

Endothelial cells within the neural crest cell migratory stream appeared close enough to be in physical contact with neural crest cells (Fig. 1b–e). We find that there is physical contact between neighboring neural crest and endothelial cells both near the neural crest migratory stream (Fig. 1b, d) and in the branchial arches (Fig. 1c, e). To examine this further in three dimensions, we fluorescently marked premigratory neural crest cells with a membrane label (DiI) (Fig. 1f) in transgenic quail embryos at HH St9 and observed the cells approximately 24 h later at HH St15 (Fig. 1g–j). The large branchial arch arteries can be seen surrounded by neural crest cells and loose endothelial cells in the branchial arch tissue (Fig. 1h). Together, these data suggest there may be complex, dynamic cell interactions between the neural crest and endothelial cells.

### Time-lapse analysis revealed a multistep process to the neural crest and endothelial cell interactions in the head

**Fig. 2:**
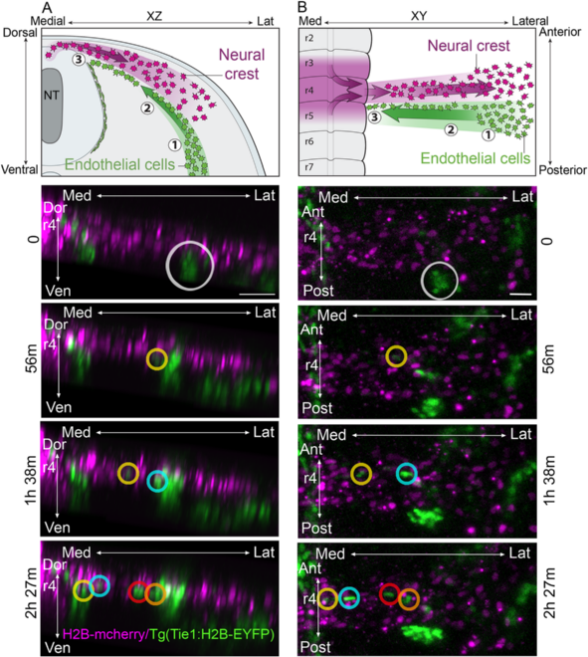
Time-lapse analysis reveals a multistep sequence to the neural crest and endothelial cell interactions in r4. Transverse (**a**) and dorsal (**b**) view of neural crest cells migrating away from the neural tube in stereotypic patterns and endothelial cells moving in the opposite direction towards the neural tube. Schematics at *top* outline the multistep sequence of migration: 1, endothelial cell cluster near the dorsal aorta; 2, as neural crest cells pass the dorsal aorta, the endothelial cells sprout and migrate alongside the neural crest stream; 3, the endothelial cells connect with the plexus around the neural tube. The images *below* are of the same embryo time-lapse images shown from a transverse (**a**) and dorsal (**b**) view at indicated elapsed times of H2B-mCherry labeled neural crest (*magenta*) and H2B-YFP endothelial cells (*green*). *White circle* (0 h 0 min) highlights visible portion of dorsal aorta where endothelial cells will sprout. *Colored circles* indicate individual endothelial cells to aid in visual tracking between frames and views of the time lapse. Scale bar 50 μm


Labeling of the premigratory cranial neural crest cells in Tie1:H2B-EYFP transgenic quail embryos with a nuclear localized H2B-mCherry fluorescent protein allowed us to visualize and track the cell dynamics of both cell populations in the same embryo (Fig. 2; Additional files 1 and 2, *n* = 10). In a typical time-lapse imaging session, we were able to record in vivo neural crest and endothelial cell dynamics with single - cell resolution. Focusing on the pre-otic r4 neural crest cell migratory stream, we observed a multistep process to neural crest and endothelial cell migration events. First, endothelial cells from the dorsal aorta became visually distinguishable as cells moved in a ventral-to-dorsal manner to directly underneath the dorsolateral neural crest migratory pathway (Fig. 2; Number 1, white circle). Endothelial cells adjacent to or in front of the neural crest migratory stream appeared as loosely connected cells that aggregated just below and in front of the lead neural crest cells. Second, individual endothelial cells sprouted from the aggregate to form single file chains that moved in the opposite direction towards the neural tube and alongside the neural crest cell migratory stream or just below (Fig. 2; Number 2, 56 min). On occasion, endothelial sprouting and chain formation appeared along both the rostral and caudal sides of the neural crest cell migratory stream (Additional file 2), but more commonly this occurred on the caudal side (Additional file 1). Third, endothelial sprouts moved ventrally to join the perineural vascular plexus surrounding the neural tube (Fig. 2; Number 3, 2 h 27 min) and maintained a single-cell-wide chain between the dorsal aorta and perineural vascular plexus. Thus, pre-otic migrating neural crest cells encountered individual migrating endothelial cells moving in the opposite direction that resulted in the segregation of the two cell populations to discrete, but spatially juxtaposed migratory pathways.

Additional file 1. Overview of r3 to r7 neural crest and endothelial cell migration. Time-lapse, confocal images of transgenic Tie1:H2B-YFP quail embryo (nuclei of endothelial cells, *green*) with premigratory neural crest labeled with DiI from r3 to r7 (*magenta*). Movie begins at roughly HH St11 after some neural crest cells have already emerged from the neural tube. Both populations of cells are highly dynamic and migrate in close spatial proximity. (MOV 6586 kb)
Additional file 2. The r4 migratory neural crest stream and endothelial cells migrate in close proximity. Time-lapse, confocal images of developing quail embryo at the r4 level of the head beginning at roughly HH St11. *Top*: dorsal view, *bottom*: transverse view of the same embryo. Nuclei of vasculature (*green*) coalesce near the dorsal aorta and sprout dorsally towards the neural tube, brushing against the neural crest stream (*magenta*). Neural crest cells pass the dorsal aorta at the same time this sprouting occurs while migrating ventrally. The endothelial cells create a chain following each other in the space just ventral to the neural crest stream, then connect to the perineural vascular plexus surrounding the neural tube. (MOV 3718 kb)


**Fig. 3:**
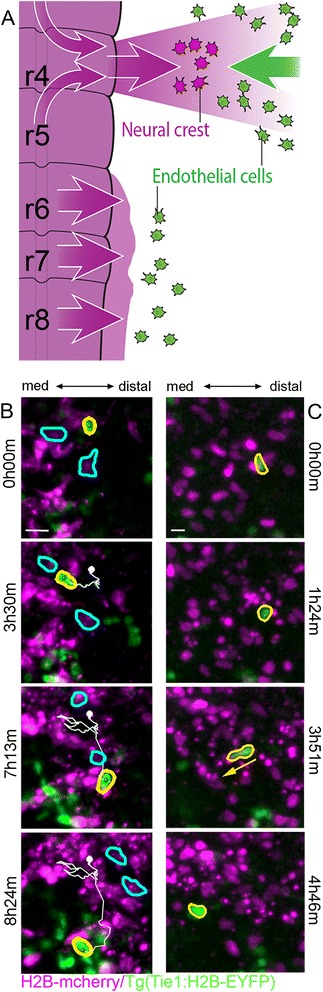
Time-lapse analysis in r6 neural crest stream shows frequent collisions with endothelial cells. **a** Schematic of embryo during time-lapse imaging illustrating the neural crest migrating away from the neural tube and endothelial cells moving in the reverse direction. **b** Example of an endothelial cell (*yellow*) that collides with the dense r6 neural crest stream, changes direction, and moves to a less populated area. Two neural crest cells (*cyan*) that interact with the endothelial cell migrate distally. **c** Endothelial nuclei (*yellow*) changing from a round to elongated shape as the cell squeezes through neural crest. Scale bars 50 μm


Visualization of post-otic neural crest and endothelial cell mixing led to surprising observations. In contrast to the r4 neural crest area, the r6 neural crest and endothelial cells move within the same migratory pathway, again in opposite directions (Fig. 3a). We saw frequent collisions between the neural crest and endothelial cells that persisted from 8 min to as long as 5 h (Fig. 3b, c; Additional files 3, 4, and 5). During these cell interactions, we observed three types of behaviors. First, endothelial cells that encountered neural crest cells moving in the opposite direction continually attempted to pursue a trajectory towards the neural tube from within the neural crest cell migratory stream (Fig. 3b; Additional file 3). After subsequent collisions, we observed the endothelial cells to change direction and move to a region of lower cell density within the neural crest cell migratory stream and resume movement towards the neural tube. Second, we repeatedly noticed that endothelial cell nuclei changed shape in order to squeeze between neighboring neural crest cells (Fig. 3b; Additional file 4). Third, some endothelial and neural crest cell collisions resulted in the movement of the endothelial cell in the reverse direction (Additional files 5 and 6). While the neural crest cells are moving distally caudal to the otic vesicle, the endothelial cell moves more slowly with the stream. This behavior persisted until the endothelial cell changed direction to move into a sparsely populated region of the neural crest cell migratory stream, and then towards the neural tube.

Additional file 3. Post-otic neural crest cells contact endothelial cells while migrating. Time-lapse confocal images of rhombomere 6 level of developing quail with endothelial cell nuclei (*green*) and neural crest (DiI, *magenta*) beginning at HH St12. Endothelial cells and neural crest in the r6 stream travel in the same plane within the embryo, resulting in collisions. Endothelial cells generally move towards the neural tube, while neural crest cells move away. The *marked cell* in the movie highlights an endothelial cell that is attempting to move through a dense patch of neural crest, cannot, then reroutes around the neural crest ventrally to a less dense region where it continues moving towards the neural tube. (MOV 6138 kb)
Additional file 4. Endothelial cells deform nuclear shape while migrating through neural crest stream. Time-lapse confocal images of rhombomere 6 level of developing quail with endothelial cell nuclei (*green*) and neural crest (H2B-cherry, *magenta*). Movie begins at HH St11. The endothelial cell is migrating towards the neural tube encountering many neural crest cells in its path that are migrating in the opposite direction. The endothelial cell nucleus greatly deforms, squeezing through the narrow gaps in the neural crest stream as it passes. (MOV 9331 kb)
Additional file 5. Endothelial cells reverse direction of migration while in dense neural crest stream. Time-lapse confocal images of rhombomere 6 level of developing quail with endothelial cell nuclei (*green*) and neural crest (H2B-cherry, *magenta*), HH St12. An endothelial cell enters a densely packed neural crest area. The endothelial cell moves in the reverse direction until it exits the neural crest stream next to the otic vesicle. The endothelial cell then resumes migration toward the neural tube. (MOV 3622 kb)


### Migrating cranial neural crest cells express angiopoietin 2

**Fig. 4:**
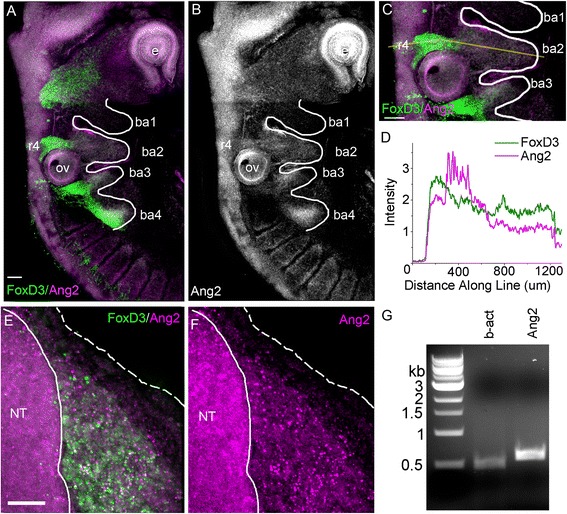
Expression of anti-angiogenic factor Ang2 by cranial neural crest cells. **a** HCR in situ image of HH St15 embryo with FoxD3 (*green*) and Ang2 (*magenta*). Scale bar 100 μm. **b** Ang2 probe only. Ang2 RNA can be seen in many locations including the eye, neural tube, cranial neural crest streams, and along the outer edge of the somites. **c** r4 neural crest stream in (**a**) with polyline drawn in *yellow* for intensity measurement. **d** Intensity profile along *yellow line* in (**c**) showing the overlap of the Ang2 and FoxD3 signals. Transverse section of HH St15 embryo at the r4 neural crest stream showing FoxD3 and Ang2 signals (**e**) and Ang2 only (**f**). Scale bar 40 μm. **g** RT-PCR of sorted r4 neural crest for beta-actin and Ang2 probes

Having previously put forward a cell-induced model of collective neural crest cell migration in which lead cells read out a guidance signal(s) and inform trailing cells to follow [39, 40], we hypothesized that there would be signals that play a role in guidance information transfer. With the presence of VEGF in the cranial neural crest microenvironment and its role in neural crest chemotaxis [14], we decided to examine the expression profile of migrating cranial neural crest cells for vascular-related genes. We discovered the expression of angiopoietin 2 (Ang2), a well-characterized vascular remodeling gene (Fig. 4). Given the previously published role of Ang2 to stimulate in vitro cell motility in many different cell types and our characterization of the unexpected encounter and complex interactions between cranial neural crest and endothelial cells within a shared migratory pathway, we hypothesize that Ang2 plays a role in cranial neural crest migration.

To analyze the in vivo mRNA expression pattern of Ang2 with higher fidelity and confirm the expression in migrating neural crest cells, we took advantage of multiplexed in situ hybridization chain reaction (HCR; [41]) and RT-PCR. HCR is a sensitive RNA imaging technique that permits cellular resolution and multiplexing such that we could visualize Ang2 mRNA expression specifically within FoxD3-labeled migrating cranial neural crest cells (Fig. 4a). We found that Ang2 is expressed by migrating cranial neural crest cells and several other tissues including the neural tube, otic vesicle, and somites (Fig. 4a, b). A transverse section through the r4 neural crest migratory stream also shows the overlap of signals between these two RNAs more clearly (Fig. 4e, f). A quantitative polyline kymograph analysis of the fluorescent signal revealed that Ang2 expression was high in the neural tube and reduced to a consistent level throughout the r4 neural crest cell migratory stream (Fig. 4c, d). To verify that the expression of Ang2 was within the r4 neural crest, we performed RT-PCR for a control gene and Ang2 on the FACS sorted r4 neural crest (Fig. 4g).

### Gain and loss of function of angiopoietin 2 in neural crest cells showed perturbations in neural crest cell migration

**Fig. 5:**
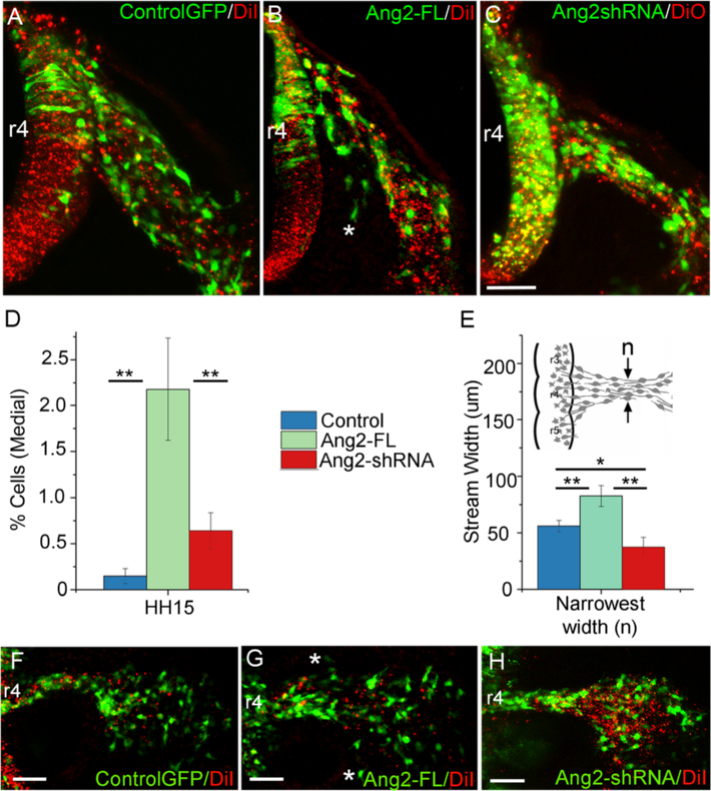
Gain and loss of function of Ang2 in neural crest cells disrupt neural crest patterning. Transverse sections of HH St15 embryo that has been injected with DiI (*red*) as well as a vector (*green*) containing GFP (**a**), Ang2-FL (**b**), or DiO and Ang2-shRNA (**c**). Neural crest can be seen in inhibitory zones at the *asterisk* in **b**. **d** Percent of total r4 neural crest cells found in atypical locations between control (*n* = 7) and Ang2-FL (*n* = 10), *p* = 0.007, and between control and Ang2-shRNA (*n* = 11), *p* = 0.06, and between Ang2-FL and Ang2-shRNA, *p* = 0.01. All error bars indicate SEM, measurements calculated per embryo. **e** Measurements on the shape of the whole neural crest stream. The width was measured above the OV at the narrowest part of the stream. Comparison between control (*n* = 12) and Ang2-FL (*n* = 10), *p* = 0.01; control and Ang2-shRNA (*n* = 8), *p* = 0.003; and Ang2-FL and Ang2-shRNA, *p* = 0.04 measurements calculated per embryo. **f**–**h** Dorsal view of whole embryo at HH St15 labeled similarly to above images. *Asterisks* indicate neural crest in prohibitory zones. Scale bars 50 μm

To examine the function of Ang2 on neural crest migration, we created an overexpression vector with the full-length Ang2 clone. When we injected and electroporated the Ang2-FL vector into premigratory chick neural crest cells, we found neural crest cells in atypical ventromedial locations, rather than along the stereotypical dorsolateral migratory pathway (Fig. 4a, b). Quantification of neural crest cell positions in control (*n* = 7) and Ang2-FL embryos (*n* = 10) showed a small but significant number of cells within ventromedial locations (Fig. 5d). To determine whether these atypical neural crest cell behaviors had an effect on the shape of the migratory stream, we measured the width of the r4 neural crest stream in control (*n* = 12) and Ang2-FL embryos (*n* = 10) at the narrowest point (Fig. 5e–g). The Ang2-FL expressing neural crest stream was wider at its narrowest spot, potentially revealing cells in the neural crest cell free zones adjacent to r3 and r5 (Fig. 5e). In addition, single r4 neural crest cells can be observed positioned rostrally and caudally to the migratory stream (Fig. 5g, asterisks).

In contrast, loss of Ang2 in premigratory neural crest cells was achieved through introduction of an shRNA designed for chick Ang2 and placed in the pRFP-RNAiC vector [32]. The shRNA vector’s ability to knock down Ang2 expression was tested by qPCR on a chick LMH cell line transfected with both gain- and loss-of-function vectors and found to have 90% knockdown (Additional file 7). In Ang2-shRNA electroporated embryos (*n* = 8), the neural crest migratory stream was significantly narrower than in controls (Fig. 5e). We did not observe aberrant neural crest cell migration into neural crest cell free zones in Ang2-shRNA embryos (*n* = 11, Fig. 5c, h). In addition, we measured the changes in stream width with the empty shRNA vector as well as control and Ang2 morpholino injection and found the neural crest stream narrows upon loss of Ang2 with either the shRNA or morpholino (Additional file 8). Together, these data suggest that neural crest cell behaviors are altered after manipulation of Ang2 to become more or less exploratory depending on gain or loss of function, respectively.

### Mean square displacement (MSD) analysis revealed neural crest cells undergo both diffusion and directed motion

**Fig. 6:**
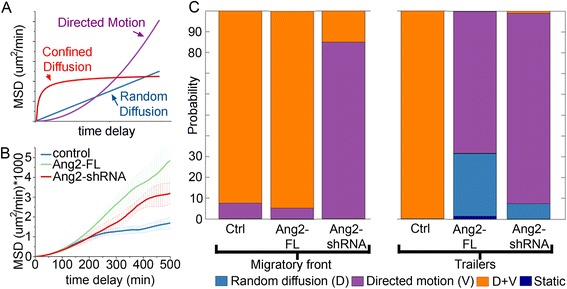
Gain and loss of Ang2 in neural crest disrupt the type of motion neural crest cells exhibit. **a** Example MSD curves for cells undergoing one type of motion during an entire time lapse. Random diffusion (*D, straight line*), directed motion (*V, parabola*), and confined diffusion (*C, asymptotic*). **b** Mean MSD curves for neural crest migrating in vivo that have been electroporated with indicated vector. Control: 10 embryos and 246 cells, Ang2-FL: 8 embryos and 118 cells, Ang2-shRNA: 4 embryos and 55 cells. While each curve is parabolic over short time intervals, the behavior deviates from a single-shaped curve over longer time intervals. **c** Bayesian analysis of in vivo neural crest MSD curves. Each cell was assigned to be in the front 30% of the stream or trailing 70%. Then the front or trailing MSD curves were analyzed by the MSD-Bayes analysis package. The probability of each type of motion is shown by the size of the bar

To analyze neural crest cell trajectories in further detail after gain or loss of function of Ang2, we performed in vivo time-lapse imaging experiments with control (*n* = 10), Ang2-FL (*n* = 8), or Ang2-shRNA (*n* = 4) electroporated neural crest and tracked single neural crest cells in the r4 migratory stream. We calculated the MSD curves for tracked cells and applied a statistical analysis to infer the type of cell motion in control, Ang2-FL, and Ang2-shRNA embryos. An MSD curve is determined by calculating the mean square displacement of a cell over different windows in time. The resulting curve is then fit to a model of cellular motion such as (D) random diffusion (straight line), (V) directed migration or flow (parabolic), (C) confined diffusion (asymptotic), or combinations of these (Fig. 6a). When we generated the MSD curves for neural crest cell trajectories in control, Ang2-FL, and Ang2-shRNA embryos, we found there was a combination of at least two types of cell motion (Fig. 6b). That is, the shape of the MSD curve was not purely a straight line, parabolic, or asymptotic. Each condition’s MSD curve is parabolic over short time intervals, but then changes shape over longer time intervals.

To better understand the MSD curve data, we implemented the MSD-Bayes MATLAB package [38]. This software is a multiple-hypothesis statistical testing technique that objectively assigns a probability of each type of motion to our calculated MSD curves. Data from our laboratory have shown that neural crest cells have different gene expression profiles and cell morphologies depending on position within a migratory stream [39, 40]. When we analyzed the MSD curves for lead cells (front 30% of the migratory stream) separately from the trailing subpopulation, we found that for cranial neural crest cells, the most probable type of motion for the lead and trailing cells was a linear combination of directed migration and diffusion (Fig. 6c, *Ctrl* columns). This made inherent sense, since we have previously observed run-and-tumble style neural crest cell behaviors in in vivo time-lapse analyses [42, 43]. To determine whether there were cell behavioral changes associated with Ang2 perturbation, we analyzed the Ang2-FL and Ang2-shRNA time-lapse data in a similar manner. Cell trajectories within the migratory front in Ang2-FL embryos resembled those in control embryos (Fig. 6c, compare the first two columns in the bar graph). However, trailing neural crest cells within Ang2-FL embryos were most likely to have very directed motion or to a lesser extent move in completely random diffusion (Fig. 6c). MSD analysis of neural crest cell trajectories in Ang2-shRNA embryos showed cells moved in a more directed manner compared to control embryos, and lost most of the diffusive behavior seen in control embryos in both the lead and trailing cell subpopulations (Fig. 6c). Together, these data suggest Ang2 overexpression had a more significant effect on trailer neural crest cell behaviors, and its knockdown resulted in increased directed motion of both leaders and trailers.

### Angiopoietin 2 exposure results in more phosphorylated focal adhesion kinase (FAK) protein in neural crest

**Fig. 7:**
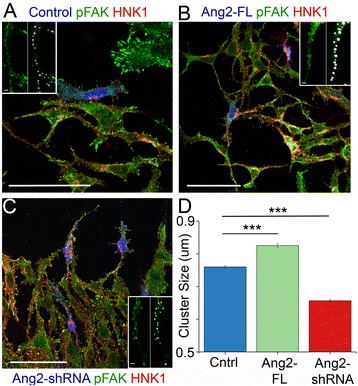
Gain and loss of Ang2 in neural crest cultures affects the amount of phosphorylated FAK. Neural tubes electroporated with either a GFP control vector (**a**), Ang2-FL (**b**), or Ang2-shRNA (**c**) were plated on fibronectin/poly-l-lysine coated glass and allowed to grow for 24 h. Electroporated vector in *blue*. The cultures were then stained with HNK1 (*red*) and pFAK (*green*) antibodies. Scale bars 50 μm. *Inset* images in **a**, **b**, and **c**: individual filopodia in the pFAK channel showing the size measurement. Scale bar 3 μm. **d** The average pFAK cluster size. Each cluster of pFAK was individually measured by the spot detection algorithm (Imaris) based on the fluorescence in the pFAK channel, so thousands of clusters were used to calculate the average, SEM, and comparison between conditions. Control 4 neural tubes and *n* = 4179 pFAK clusters, Ang2-FL 4 neural tubes and *n* = 3182 pFAK clusters, 4 neural tubes and Ang2-shRNA *n* = 3298 pFAK clusters. All conditions are statistically different from each other with control vs. Ang2-FL *p* = 1 × 10^–22^, control vs. Ang2-shRNA *p* = 5 × 10^–85^, and Ang2-FL vs. Ang2-shRNA *p* = 1 × 10^–132^

Previous work has shown that Ang2 is able to bind to specific integrin pairs on the surface of non-endothelial cell types. After Ang2 is bound to an integrin pair, the intracellular side of the integrin phosphorylates either FAK or mitogen-activated protein kinase (MAPK) [17–20]. Therefore, we performed immunohistochemistry for phosphorylated FAK at Tyr861 (pFAK) on neural tubes that had been electroporated with a control vector, Ang2-FL, or Ang2-shRNA (Fig. 7a, b, and c, respectively). All neural tubes were plated on a surface coated with fibronectin and poly-l-lysine, and neural crest cells migrated in all cases. The pFAK labeling appeared punctate as there were pFAK proteins scattered within each cell; some of the bright clusters of pFAK were small (<0.5 μm), and others were almost 1 μm in diameter (Fig. 7, insets). The sizes of the clusters visible in the migrating, HNK1-positive cells that could clearly be segmented were measured (Fig. 7d). We found that the average size of a pFAK cluster increased even when only a subset of the cells were overexpressing Ang2. The larger clusters of pFAK would indicate certain areas on the membrane of the cell are phosphorylating more FAK. In Ang2-shRNA embryos, pFAK clusters were significantly smaller (Fig. 7d). In summary, these data show that an overabundance of Ang2 results in an enhanced diffusive cell behavior, increased pFAK activity, and invasion of neural crest cells into atypical regions.

### Angiopoietin 2 gain and loss of function by cranial neural crest cells affected the migratory pattern and model of cellular motion of endothelial cells

**Fig. 8:**
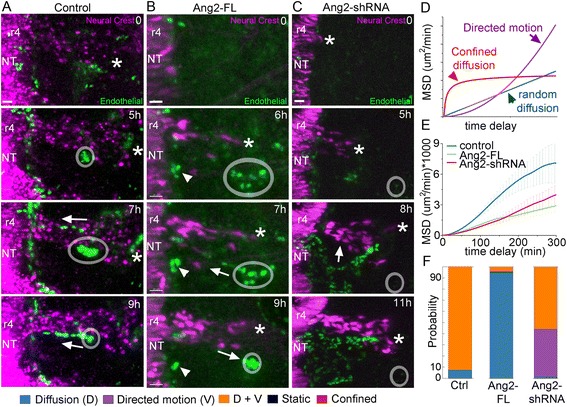
Ang2 gain and loss of function in neural crest alters endothelial cell migration. Stills from time-lapse movies of Tie1:H2B-YFP quail embryos where the premigratory neural crest has been electroporated with control vector (**a**), Ang2-FL (**b**), or Ang2-shRNA (**c**). Lead neural crest cell marked with *asterisk* in all images except control at 9 h, where the front of the stream has migrated past the image area. Endothelial cells cluster near the dorsal aorta (*white circle*) and sprout after the lead neural crest passes the dorsal aorta in control. This event occurs early in Ang2-FL. In addition, the sprout created in the Ang2-FL embryo collapses and retreats back to the dorsal aorta before it reaches the perineural vascular plexus (*triangle*). **c** Without Ang2 expression, the lead neural crest cells reach their position near the dorsal aorta, but no endothelial cells sprout. In this example, endothelial cells from below the otic vesicle emerge in a plexus to the posterior side of the neural crest stream (*white arrow*). In addition, some endothelial cells invade the stream, disrupting the neural crest migration. Isolated neural crest marked with *asterisk*. Scale bars 20 μm. **d** Example MSD curves. **e** Mean MSD curves for endothelial cells from same movies in Fig. 6. Control: 14 cells, Ang2-FL: 46 cells, and Ang2-shRNA: 14 cells. **f** Bayesian analysis of in vivo endothelial cells’ MSD curves, legend at bottom


Time-lapse imaging of cranial neural crest cells expressing control, Ang2-FL, or Ang2-shRNA revealed that the normal endothelial cell migratory pattern was disrupted. Specifically, we found that when neural crest cells are overexpressing Ang2, endothelial cells sprouted earlier from the dorsal aorta compared to wild-type embryos and prior to the neural crest cells migrating above the dorsal aorta (compare Fig. 8a and b, Additional file 9). Furthermore, premature endothelial sprouts from the dorsal aorta were not stable. We observed that premature endothelial sprouts that formed and connected with endothelial cell sprouts from the perineural vascular plexus regressed back to the dorsal aorta over time in Ang2-FL embryos (Fig. 8b, Additional file 9). In contrast, we observed a reduction in sprouting of this same vessel in Ang2-shRNA embryos (Fig. 8c, Additional file 10). In half of the movies, the endothelial cell sprouting from the dorsal aorta did not occur, but the remaining vascular pattern looked normal. However, in the other half of the time-lapse imaging sequences, we found that the vasculature moved in a large plexus from underneath the otic vesicle to eventually occupy the space along the r4 neural crest migratory stream boundary (Fig. 8c, Additional file 10). These endothelial cell movements resemble an interconnected ensemble of cells like a sheet, moving through the tissue. Time-lapse imaging also showed that a few endothelial cells moved into the r4 neural crest migratory stream and disrupted normal migration. A more detailed analysis of the endothelial cell behaviors by the MSD analysis revealed that endothelial cells also do not follow a single model of cell motility but a combination of types of motion (Fig. 8e). The MSD-Bayes analysis of the endothelial cells results in a linear combination of diffusion and directed motion, similar to neural crest cells, in wild-type embryos (Fig. 8f). Strikingly, endothelial cells show a predominantly random diffusive cell behavior in Ang2-FL embryos. In contrast, endothelial cells displayed a higher probability of directed motion in Ang2-shRNA embryos.

Additional file 9. Gain of Ang2 expression in neural crest cells alters timing and pattern of endothelial cell migration. Time-lapse, confocal images of developing quail embryo at the rhombomere 4 level of the head beginning HH St10. The neural crest cells, labeled with an overexpression vector for Ang2 (*magenta*), migrate away from the neural tube while endothelial cells (*green*) sprout from the dorsal aorta and migrate in the reverse direction. In general, as the neural crest passes the dorsal aorta, the endothelial cells sprout. However, with the overexpression of Ang2, the endothelial cells sprout earlier from the dorsal aorta and form a multicellular chain, but the chain collapses back to the dorsal aorta. (MOV 8304 kb)
Additional file 10. Loss of Ang2 expression in neural crest creates new vascular plexus on caudal side of neural crest stream. Time-lapse, confocal images of developing quail embryo at the rhombomere 4 level of the head beginning HH St10. The neural crest cells are marked with a vector containing an Ang2-shRNA in *magenta* migrating away from the neural tube, while endothelial cells (*green*) form an unusual pattern emerging from below the otic vesicle. Normally a sprout of endothelial cells is seen extending from the dorsal aorta and moves along the caudal side of the r4 stream, but in this embryo where Ang2 is not expressed in the neural crest, a plexus of endothelial cells moves to the caudal side of the r4 neural crest stream. In addition, a multicellular structure of endothelial cells pushes into the neural crest stream, causing a few leading neural crest cells to become separated from the stream. These cells halt their forward progression until contact is made with the stream again. (MOV 9067 kb)


## Discussion

We used the quail model system to study the role of angiopoietin 2 (Ang2) in cranial neural crest cell migration. Gene profiling in our previous study identified Ang2 expression by microarray in migrating chick cranial neural crest cells; however, its role was unclear. Ang2 is a secreted factor and has been shown to stimulate cell migration in vitro in a variety of non-endothelial cell types [17–21]. Given this evidence, we hypothesized that Ang2 functions to stimulate collective neural crest cell migration or invasiveness. Furthermore, Ang2 has been studied for its role in destabilizing endothelial cell junctions, and in the presence of VEGF or other growth factors, it can induce sprouting of disassembled endothelial cells [15, 44, 45]. Previous static analyses in mouse [46] and chick [4, 47] hinted at a potential dynamic interplay between migrating neural crest and endothelial cells during head patterning. By combining cell labeling, molecular perturbation, and time-lapse microscopy within a Tie1:H2B-YFP transgenic quail embryo, we were able to address our hypothesis and describe novel aspects about the spatio-temporal dynamics of neural crest and endothelial cells in the same embryo.

Static analyses of quail developmental stages showed the progression of cranial neural crest and endothelial cell positions and their close proximity, strengthening the argument for a dynamic interplay and cell sorting process during head patterning. By using the combination of the transgenic Tie1:H2B-YFP quail, neural crest cell labeling, and tissue clearing, we were able to more closely examine the spatial distribution of the endothelial cells near the neural crest cell migratory streams without relying on a perfusion technique that would have missed smaller, non-patent vessels or single cells undergoing vasculogenesis. At all stages examined (HH St9–15), at least a few endothelial cells were approximately 20 μm or closer to the r4 neural crest cell migratory stream. 3D reconstruction of images from collected z-stacks in tissue-cleared quail embryos showed how the neural crest cells surround the branchial arch arteries and the anatomical relationship between the neural crest cell migratory stream and endothelial cells that move to the perineural vascular plexus. Thus, although arising in distinct locations for the most part, neural crest and endothelial cells appeared to have a symbiotic relationship along a common migratory pathway prior to assembling structures within the peripheral branchial arches.

Time-lapse analysis revealed a multistep process to neural crest and endothelial cell interactions, suggesting a stereotypical and coordinated patterning mechanism. During a typical time-lapse imaging session, individual endothelial cells were observed to initially gather near the dorsal aorta and sprout in single file chains towards the dorsal neural crest cell migratory pathway, immediately after the migratory front of the r4 neural crest cell migratory stream passed above. Endothelial cells then migrated alongside the rostro-caudal borders and underneath the neural crest cell migratory stream and in the opposite direction to connect with the perineural vascular plexus surrounding the neural tube. Given the correlation in time of the passing of the r4 neural crest cell migratory stream directly above the disassembly and the sprouting of the endothelial cells from the dorsal aorta, it is possible to speculate this event is coordinated. Furthermore, the expression of Ang2 by migrating neural crest cells and the presence of VEGF in the surface ectoderm and BA2 may provide a mechanistic basis for further study of how the neural crest and endothelial cells coordinate movements. In contrast to the pre-otic region, post-otic neural crest and endothelial cell interactions were less coordinated. The frequency of the two cell populations bumping into each other and subsequent changes in endothelial cell trajectories suggested a coarse molecular choreography dictated by the neural crest. Subsequent studies may uncover different molecular signals directing cell trafficking in this subregion of the embryo.

Knowledge of the spatio-temporal expression pattern of Ang2 helped us determine whether Ang2 has a functional role on lead neural crest cells within the migratory front or on cells throughout the stream. Multiplexed fluorescence HCR confirmed the expression correlation between Ang2 and FoxD3, marking the migrating neural crest cells (Fig. 4). The quantitative polyline kymograph analysis of the fluorescence signals showed expression of Ang2 throughout the neural crest cell migratory stream at HH St13 (data not shown) and HH St15 (Fig. 4). This expression pattern suggests that Ang2 protein is produced by both lead and trailing subpopulations of the cranial neural crest throughout migration and suggests that Ang2 functions to stimulate invasion of the neural crest stream rather than direct cells. Given our static and time-lapse data revealing the detailed interplay of neural crest and endothelial cells, it makes logical sense to speculate that Ang2 functions to ensure endothelial cells do not prematurely organize into stable blood vessels that would occlude neural crest migration. Alternatively, changes in cranial neural crest cell migration could affect endothelial cell dynamics by introducing structural changes in the microenvironment or an absence of other signaling molecules from the migrating neural crest that would affect endothelial cell behaviors. Further gene profiling of the cranial neural crest and endothelial cells during the sequence of developmental stages of migration may reveal the broader spectrum of molecular choreography between these two cell populations.

Perturbation of Ang2 signaling in neural crest cells led to changes in cell behavior, cell invasion into typical neural crest cell free zones, and increased FAK phosphorylation, suggesting a critical role for Ang2 in collective neural crest cell migration. In the embryo, gain of Ang2 function caused expansion of the neural crest cell migratory stream into neighboring inhibitory regions as well as individual cell invasion 100 μm or more away from the collective group and into the ventral paraxial mesoderm (Fig. 5). MSD analysis revealed that trailing neural crest cells responded differently than lead cells to gain of Ang2 function by splitting their behavior between strictly following a trail (directed) and complete exploratory motion (random). That is, trailing neural crest cells typically display a follow-the-leader behavior and maintain spatial order with respect to lead cells [48], rather than the exploratory behavior we observed after gain of Ang2 function (Fig. 5). This would imply that the cells which moved away from the typical stream were trailing neural crest cells.

Loss of Ang2 function led to constriction of the neural crest cell migratory stream and loss of random motion or exploratory behavior. Given this trailing cell behavior evidence, it is interesting to speculate that Ang2 functions to promote cell invasion by enhancing chemokinesis or exploration. This would agree with our previous time-lapse imaging data that showed trailing neural crest cells move with a higher speed but less directionality than leaders [42, 43]. Maintaining trailing neural crest cells in a chemokinetic rather than sustained directed motion would have a twofold purpose. First, the diffusive behavior of trailing neural crest cells would allow frequent neighbor exchanges so as to maximize the ability of cells to find other neighbors with guidance information. Although trailing cells are more diffusive, this would also ensure that the lead cells would not be leap-frogged by trailing cells, as has been observed in the gut [49]. This spatial order of collective neural crest cell migration promoted by Ang2 in the head also agrees with our previous data indicating that cranial neural crest cells migrate in a spatially ordered manner [48]. Second, the diffusive behavior of trailing neural crest cells would allow cells to be more responsive to dynamic changes in guidance signals. That is, trailing neural crest cells in both the head and trunk need to respond to guidance signals that direct cells into proximal targets such as contributing to the cranial ganglia or dorsal root ganglia, respectively. Thus, it makes logical sense that trailing neural crest cells maintain a diffusive behavior that is regulated by Ang2 signaling in order to promote collective cell migration.

## Conclusions

To our knowledge, these data represent the first report of the critical function of Ang2 during collective neural crest cell migration and detailed characterization of the spatio-temporal dynamics of neural crest and endothelial cells during vertebrate head patterning. We propose a model in which Ang2 is secreted by migrating neural crest cells and stimulates cell invasiveness. Our hypothesis of the role of Ang2 to promote neural crest migration supported by these results fits well with our previously proposed cell-induced gradient model [39, 40] in which lead cells read out guidance cues and transfer information to trailing cells. We suggest that by regulating Ang2 activity, cranial neural crest cells move collectively in a stream by directed migration of leaders and diffusive behavior of trailing cells. Furthermore, the idea of migrating cranial neural crest cells that display dynamic cell-cell interactions with endothelial cells and secrete a vessel destabilization ligand fuels the speculation that signals from the neural crest direct the timing and migratory pattern of endothelial cells. Given that we show the timing of the cranial neural crest cell migratory passes  directly above the dorsal aorta, Ang2 expression may offer a molecular in road to dissect further signals that stimulate endothelial cells to disassemble and respond to migrate towards VEGF sources in the surface ectoderm and branchial arches. Future studies should shed light on what microenvironmental signals regulate Ang2 expression and how its expression is timed to control neural crest cell behaviors and the intimate relationship between the neural crest and endothelial cells during head patterning.

## Additional files

Additional file 6: Time-lapse analysis in r6 neural crest stream shows endothelial cell moving in reverse direction. An endothelial cell enters a dense neural crest stream caudally to the otic vesicle (*OV*) and begins moving with the stream instead of proximally. After exiting the stream, the cell continues with its normal migration proximally. Scale bar 50 μm. (TIF 699 kb)
Additional file 7 Chick LMH cell line was transfected with a GFP, an Ang2-FL, or a combination of Ang2-FL and Ang2-shRNA vectors. RNA was harvested from cultures 24 h later and qPCR performed for Ang2 expression as well as two reference genes, GAPDH and RhoA. Expression of Ang2 in each sample with four technical replicates is plotted relative to Ang2-FL. Error bars represent SEM. (TIF 200 kb)
Additional file 8: Loss of function of Ang2 in neural crest cells creates a thinner stream of neural crest by shRNA or morpholino. (A) The width of the neural crest stream was measured above the OV at the narrowest part of the stream. Comparison between GFP control (*n* = 12) and Ang2-FL (*n* = 10), *p* = 0.01, control RNAi, the empty pRFP-RNAiC vector (*n* = 6), and Ang2-shRNA (*n* = 8), *p* = 0.03, and a scramble morpholino (*n* = 15) and Ang2 morpholino (*n* = 19), *p* = 0.01. *Error bars* indicate SEM. Additional Ang2 resulted in a wider stream, and knockdown of Ang2 by either shRNA or morpholino created a narrower stream. (B) Schematic of location of measurement. (C–H) Dorsal view of whole embryo at HH St15 with indicated vectors, dye, or morpholino. Scale bars 50 μm. (TIF 2972 kb)
