# Supplementary figures and images for: Angiopoietin 2 signaling plays a critical role in neural crest cell migration
Source: BMC Biol. 2016 Dec 15;14:111. doi: 10.1186/s12915-016-0323-9 (PMC5159958; doi:10.1186/s12915-016-0323-9)

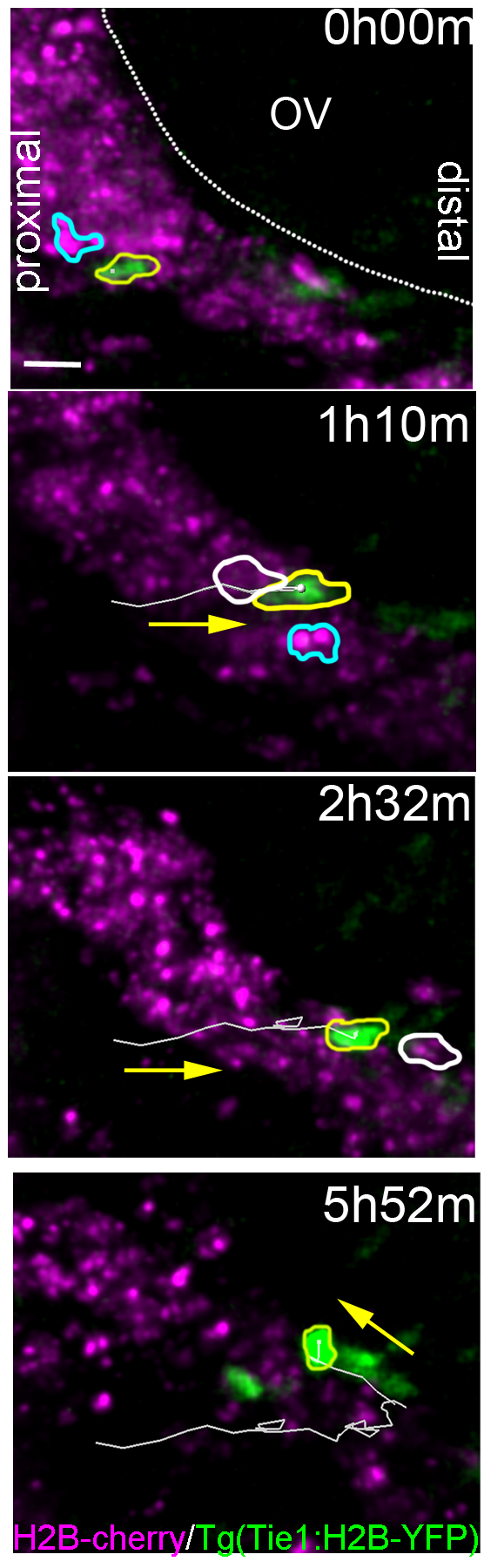

Supplement: Additional file 6: — Time-lapse analysis in r6 neural crest stream shows endothelial cell moving in reverse direction. An endothelial cell enters a dense neural crest stream caudally to the otic vesicle (OV) and begins moving with the stream instead of proximally. After exiting the stream, the cell continues with its normal migration proximally. Scale bar 50 μm. (TIF 699 kb) [file 12915_2016_323_MOESM6_ESM.tif]

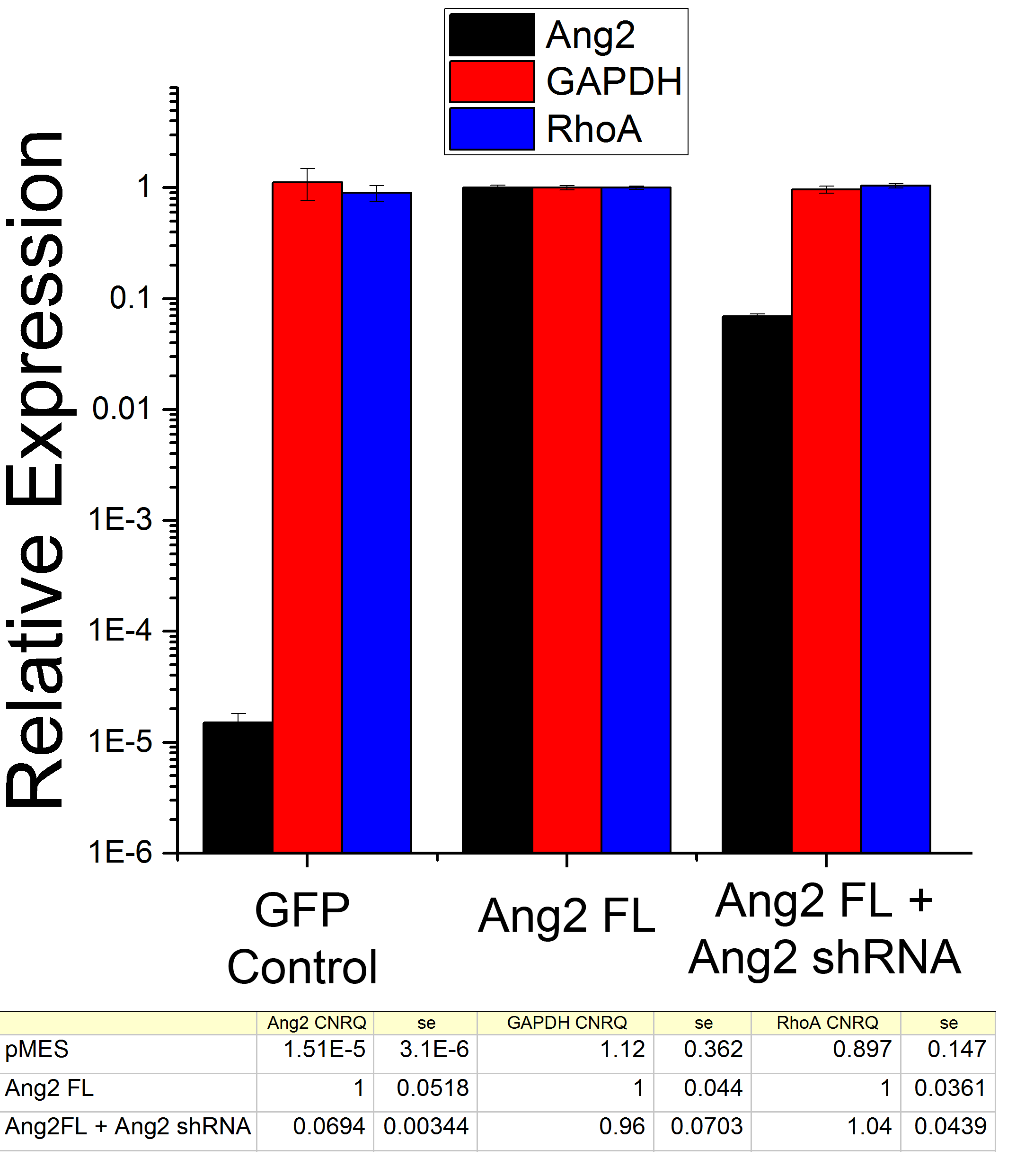

Supplement: Additional file 7 — Chick LMH cell line was transfected with a GFP, an Ang2-FL, or a combination of Ang2-FL and Ang2-shRNA vectors. RNA was harvested from cultures 24 h later and qPCR performed for Ang2 expression as well as two reference genes, GAPDH and RhoA. Expression of Ang2 in each sample with four technical replicates is plotted relative to Ang2-FL. Error bars represent SEM. (TIF 200 kb) [file 12915_2016_323_MOESM7_ESM.tif]

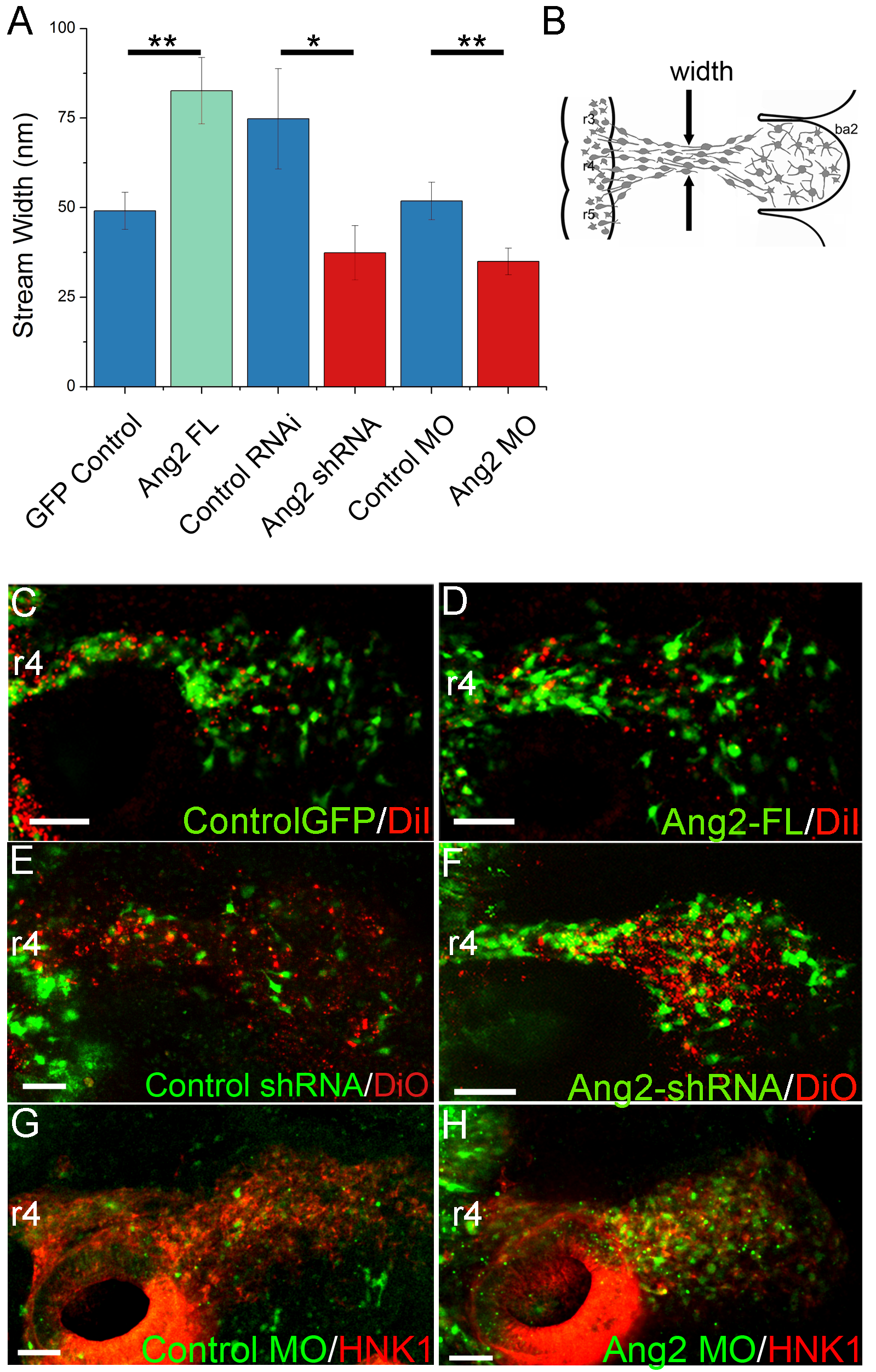

Supplement: Additional file 8: — Loss of function of Ang2 in neural crest cells creates a thinner stream of neural crest by shRNA or morpholino. (A) The width of the neural crest stream was measured above the OV at the narrowest part of the stream. Comparison between GFP control (n = 12) and Ang2-FL (n = 10), p = 0.01, control RNAi, the empty pRFP-RNAiC vector (n = 6), and Ang2-shRNA (n = 8), p = 0.03, and a scramble morpholino (n = 15) and Ang2 morpholino (n = 19), p = 0.01. Error bars indicate SEM. Additional Ang2 resulted in a wider stream, and knockdown of Ang2 by either shRNA or morpholino created a narrower stream. (B) Schematic of location of measurement. (C–H) Dorsal view of whole embryo at HH St15 with indicated vectors, dye, or morpholino. Scale bars 50 μm. (TIF 2972 kb) [file 12915_2016_323_MOESM8_ESM.tif]
